# The Impact of Anti-rheumatic Drugs on the Seroprevalence of Anti-SARS-CoV-2 Antibodies in a Cohort of Patients With Inflammatory Arthritis: The MAINSTREAM Study

**DOI:** 10.3389/fmed.2022.850858

**Published:** 2022-03-11

**Authors:** Ennio Giulio Favalli, Andrea Gobbini, Mauro Bombaci, Gabriella Maioli, Martina Biggioggero, Elisa Pesce, Andrea Favalli, Martina Martinovic, Tanya Fabbris, Edoardo Marchisio, Alessandra Bandera, Andrea Gori, Sergio Abrignani, Renata Grifantini, Roberto Caporali

**Affiliations:** ^1^Division of Clinical Rheumatology, ASST Gaetano Pini-CTO Institute, Milan, Italy; ^2^Department of Clinical Sciences and Community Health, Research Center for Adult and Pediatric Rheumatic Diseases, Università degli Studi di Milano, Milan, Italy; ^3^Istituto Nazionale Genetica Molecolare, Padiglione Romeo ed Enrica Invernizzi, Milan, Italy; ^4^Dia.Pro, Diagnostic Bioprobes Srl, Milan, Italy; ^5^Infectious Diseases Unit, Fondazione IRCCS Ca' Granda Ospedale Maggiore Policlinico, Milan, Italy; ^6^Department of Pathophysiology and Transplantation, Università degli Studi di Milano, Milan, Italy; ^7^Centre for Multidisciplinary Research in Health Science (MACH), Università degli Studi di Milano, Milan, Italy

**Keywords:** seroprevalence, SARS-CoV-2, humoral response, rheumatic musculoskeletal diseases, disease-modifying anti-rheumatic drugs, risk of infection, COVID-19

## Abstract

**Objectives:**

Given the high occurrence of asymptomatic subsets, the true prevalence of SARS-CoV-2 infection in rheumatic patients is still underestimated. This study aims to evaluate the seroprevalence of SARS-CoV-2 antibodies in rheumatic musculoskeletal diseases (RMD) patients receiving immunomodulatory drugs.

**Methods:**

All consecutive patients with rheumatoid arthritis or spondyloarthritis receiving disease-modifying antirheumatic drugs (DMARDs) evaluated between 4th May and 16th June 2020 were included. All participants were tested for anti-SARS-CoV-2 antibodies (IgG, IgM, IgA) by ELISA and were questioned about previous COVID-19 symptoms and clinical course. Results were compared with healthy population from the same region and with a control group of healthy subjects diagnosed with confirmed COVID-19.

**Results:**

The study population includes 358 patients. The overall prevalence of anti-SARS-CoV-2 antibodies (18.4%) was higher than prevalence rate based on swab-positivity (1.12%) or clinically suspected cases (10.6%), but consistent with seroprevalence observed in the healthy population. Among seropositive patients 58% were asymptomatic. Mean anti-SARS-CoV-2 titer was comparable with the control group. No differences in seroprevalence were observed according to age, sex, rheumatic disease and treatment with conventional, biologic or targeted synthetic DMARDs, whereas glucocorticoids and comorbidities resulted in higher seroprevalence rate.

**Conclusions:**

The results of this study are reassuring about the low impact of RMDs and immunomodulatory therapies on the risk and clinical course of COVID-19 and on humoral immune response to SARS-CoV-2 infection.

## Introduction

SARS-CoV-2 infection, which exploded as a pandemic during 2020 causing over three million deaths worldwide, has an extremely variable spectrum of clinical presentation from pauci-symptomatic flu-like subsets to a critical disease with respiratory failure and multiorgan dysfunction, potentially resulting in death (1). In this scenario, it is imperative to clarify whether patients with rheumatic musculoskeletal diseases (RMDs) carry a higher risk of contracting Coronavirus Disease 2019 (COVID-19) or experience a more severe course of infection than the general population (2).

Several epidemiological studies based on the observation of infectious symptoms and nasopharyngeal swab (NPS) positivity have demonstrated a similar prevalence of COVID-19 in rheumatic patients and healthy population, suggesting that RMDs and their immunosuppressive treatment do not confer an increased susceptibility to infection (3–6). However, NPS is inaccurate in detecting the disease with a false-negativity rate of at least 20% (7), and a very large proportion of subjects have an asymptomatic infection that makes them escape common diagnostic procedures (8). Thus, studies based on the seroprevalence of anti-SARS-CoV-2 antibodies can decisively contribute to clarify the real spread of COVID-19 (9, 10). To date, such data in RMD patients are still very limited (11, 12), leaving many questions open that are essential for the proper management of these fragile patients. In addition, insights into the humoral response of RMD patients are of critical interest to optimize vaccination strategies.

To fill this gap, we conducted an observational seroprevalence study in a cohort of inflammatory arthritis patients receiving immunomodulatory drugs.

## Methods

### Study Population

The study included all consecutive patients aged ≥18 years and living in Lombardy, diagnosed with rheumatoid arthritis (RA) or spondyloarthritis (SpA), with a follow-up visit scheduled between 4th May and 16th June 2020 at the outpatient rheumatology clinic of ASST Gaetano Pini-CTO Institute in Milan, a tertiary referral medical institution. All patients were receiving disease-modifying antirheumatic drugs (DMARDs) including conventional synthetic DMARDs (csDMARDs) (methotrexate, leflunomide, sulfasalazine, hydroxychloroquine, cyclosporin), biologic DMARDs (bDMARDs) (anti-TNFα mAbs; anti-IL-6R mAbs: CTLA-4-Ig; anti-IL 23 mAb; anti-IL 17A mAbs: anti-CD 20 mAb; IL1-RA/anti-IL 1β) or targeted synthetic (tsDMARDs) (Janus kinase inhibitors or PDE4 inhibitor), alone or in combination. The analysis was approved by the Ethics Committee Milano Area 2. All included patients signed an informed consent to participate in the study. A cohort of healthy residents in Lombardy (*n* = 64.660) included in a nationwide seroprevalence study conducted by the National Institute of Statistics (ISTAT) during the same timeframe was used as a control group (external control group) for seroprevalence comparison (13). In addition, a group of non-RMD subjects (*n* = 13) recovered from mild-to-moderate COVID-19 confirmed by NPS was used as a comparator for anti-SARS-CoV-2 antibody titer analysis.

### Clinical Data Collection

Demographic and clinical data were collected during the scheduled visit. In addition, with regard to the period between the onset of the pandemic in Lombardy (25th February 2020) and the visit, we recorded the occurrence of signs/symptoms suggestive of COVID-19; a potential diagnosis of COVID-19 based on NPS; any admission to ordinary hospital or intensive care unit (ICU) because of COVID-19; any close contact with established COVID-19 cases; the maintenance of usual rheumatological therapy throughout the selected period. We defined patients with positive RT-PCR NPS as *confirmed COVID-19*, whereas patients who developed respiratory symptoms compatible with a mild viral infection but had no access to NPS were defined as *highly suspicious COVID-19*, according to the relevance of registered signs/symptoms as reported in Supplementary Table 1 (two major signs/symptoms or one major sign/symptom plus at least two minor signs/symptoms).

### Serology Testing

Sera obtained from both the participants and the internal control group were processed by using ELISA assays-based tests from Diapro (Milan, Italy). We tested IgG, IgM and IgA against SARS-CoV-2 receptor binding domain (RBD) and nucleocapsid (N), whose detection is more sensitive than spike subunits (S1/S2) (14). According to manufacturer's instructions, the cut-off for the definition of a positive sample was set at S/Co > 1.5. In the ISTAT control group seroprevalence was determined as positivity for anti-N IgG (by Abbott Architect anti-SARS-CoV-2 ELISA kit).

### Statistical Analysis

The comparative analysis of anti-SARS-CoV-2 titer vs. the internal control group and the search for factors associated with SARS-CoV-2 IgG positivity was conducted only on anti-RBD which has been shown to provide the best specificity for SARS-CoV-2 with limited cross-reactivity with other coronaviruses. Differences between subgroups were analyzed by a chi-squared test for categorical variables. The comparison between different subgroups was analyzed with Kruskal-Wallis test or Mann-Whitney test. Comparison of anti-N IgG seroprevalence with the ISTAT cohort was performed by a chi-square test. Statistical analyses were performed using SPSS statistical software, version 20.0 (SPSS, Chicago, IL, USA). *P*-values equal to or < 0.05 were considered statistically significant.

## Results

### Study Population

The study population included 358 patients diagnosed with RA (*n* = 200, 56%) or SpA (*n* = 158, 44%). Mean age was 54.2 (± 13.9) years, and 64% were female. Age and gender distribution were as expected according to the specific type of arthritis.

All patients were on stable treatment with DMARDs for at least 6 months, comprising b/tsDMARDs (*N* = 300), alone or in combination with conventional treatment, and csDMARDs alone (*N* = 58) (mainly methotrexate) (Table 1). Among targeted therapies, anti-tumor necrosis factor (TNFα), anti-interleukin-6 receptor (IL6R) and Cytotoxic T-lymphocyte associated antigen-4 immunoglobulin fusion proteins (CTLA4-Ig) were the most commonly used (48.3, 11.7, and 9.8%, respectively). Approximately one-third of the patients were on concurrent chronic treatment with glucocorticoids (GC, mean dose 4 mg daily, prednisone equivalent). The majority of enrolled patients had a long-term established disease (median disease duration 15 years).

**Table 1:** Characteristics of the study population.

	**RMD patients**	**RA**	**SpA**	**Non-RMD control group**
	***n* = 358**	***n* = 200**	***n* = 158**	***n* = 12**
Age, mean (SD), years	54.2 (13.9)	57.6 (12.9)	49.8 (13.2)	45.6 (18.8)
Female, *n* (%)	230 (64.2)	155 (77.5)	75 (47.5)	7 (58.3)
Comorbidities	126 (35.3)	81 (40.5)	45 (28.6)	4 (33.3)
Disease duration, median (SD), years	15.28 (10.5)	14 (10.7)	17.2 (9.9)	-
Anti-rheumatic treatment				
bDMARDs (%)	**278 (77.6)**	**141 (70.5)**	**137 (86.7)**	
Anti-TNF	173	61	113	-
Abatacept	42	39	3	-
Anti-IL 6	35	34	1	-
Anti-IL 17	14	0	14	-
Rituximab	6	6	0	-
Anti-IL 1	3	1	2	-
Anti-IL 12/23	4	0	4	-
tsDMARDs (%)	**22 (6.2)**	**16 (8)**	**6 (3.8)**	**-**
JAKi	17	16	1	-
PDE4i	5	0	5	-
csDMARDs association (%)	**139 (38.9)**	**93 (46.5)**	**46 (29.3)**	**-**
Methotrexate	112	75	37	-
Leflunomide	11	8	3	-
HCQ	21	21	0	-
Cyclosporine	2	0	2	-
Sulfasalazine	3	0	3	-
csDMARDs mono (%)	**58 (16.24)**	**43 (21.5)**	**15 (5.8)**	**-**
Methotrexate	42	31	10	-
Leflunomide	1	0	1	-
HCQ	12	11	1	-
Sulfasalazine	2	0	2	-
Prednisone (%)–avarage dose	**105 (29.4)−4 mg**	**80 (40)−3.9 mg**	**25 (15.9)−4.3 mg**	**-**

*RA, rheumatoid arthritis; SpA, spondyloarthritis; HCQ, hydroxychloroquine; csDMARDs, conventional synthetic disease-modifying anti-rheumatic drugs; b/tsDMARDs, biological/targeted synthetic disease-modifying anti-rheumatic drugs; TNF, tumor necrosis factor; IL, interleukin; JAKi, Janus kinase inhibitors; PDE4i, phosphodiesterase 4 inhibitor. The bold values refer to the overall seropositivity result*.

Throughout the study period, 38 patients were defined as highly suspicious COVID-19, four as confirmed COVID-19, 33 reported close contacts with established COVID-19 cases. The most frequently reported symptoms were cough (12.8%), asthenia (12.5%), fever (10.9%), and ageusia/anosmia (4.5%). Five patients (four confirmed COVID-19 and one with negative NPS) required hospitalization with low-flow oxygen supplementation. No patient has been admitted to ICU and no deaths have been reported (Supplementary Figure 1).

### Seroprevalence of SARS-CoV-2 Infection and Clinical Correlation

Antibody specificity data are reported in Table 2. Briefly, 66 (18.4%) out of 358 patients tested positive for at least one anti-SARS-CoV-2 antibody (IgG/IgM/IgA, anti-RBD/N), compared with four out of 358 patients (1.12%) with swab-confirmed COVID-19 (*p* < 0.0001). Most seropositive patients tested positive for a single antibody class (37/66, 56%), while one-third tested triple positivity (IgG+IgM+IgA). Among patients who tested positive to serological test, the majority (57.5%) reported no symptoms in the 2 months prior to the blood draw. Ten patients (15.4%) referred one or two symptoms, 8 (12.3%) three symptoms, 9 (13.8%) four or more symptoms. The most frequently reported symptoms were cough (23%), fever (18.5%), asthenia (18.5%), and smell or taste loss (15.4%). Four patients (6%) were hospitalized (Supplementary Figure 1). The difference in prevalence rate (seroprevalence vs. swab-confirmed cases) confirms that the spread of COVID-19 among the population is much higher than that clinically observed. At the same time, the detection of high fraction of asymptomatic cases among RMD patients under immunosuppressant therapies is quietly reassuring about the severity and clinical outcomes of COVID-19 in this population.

**Table 2:** Seroprevalence of anti-SARS-CoV-2 antibody classes and specificities.

**Antibody class**	**Positive cases (*n*)**	**Seroprevalence (95% CI)**
IgG	29	8.1% (5.4–11.7)
IgG anti-N	28	7.8% (5.2–11.3)
IgG anti-RBD	21	5.9% (3.6–8.9)
IgM	41	11.5% (8.2–15.6)
IgM anti-N	29	8.1% (5.4–11.7)
IgM anti-RBD	27	7.5% (5–11)
IgA	42	11.7% (8.5–16)
IgA anti-N	30	8.4% (5.7–12)
IgA anti-RBD	21	5.9% (3.6–8.9)
Anti-RBD	33	9.2% (6.3–13)
Anti-N	55	15% (11.6–20)
At least 1 (IgG/IgM/IgA)	**66**	**18.4% (14–23.2)**

### Comparison With General Population

To compare the seroprevalence rate of SARS-CoV-2 between RMD patients and general population, we extrapolated data from the ISTAT survey (13), where the seroprevalence was defined by the detection of IgG against N protein. In our cohort, selecting the subjects positive for IgG anti-N, the seroprevalence rate was 7.8% (95% CI 5.2–11.3), thus comparable to that estimated in the general population resident in Lombardy region in the same period, that was 7.5% (95% CI 6.8–8.3%) (*p* = 0.50), with a similar rate of asymptomatic infections (28.5 vs. 27.3%, respectively; *p* = 0.81).

### Magnitude of Immune Response

**Figure 1:**
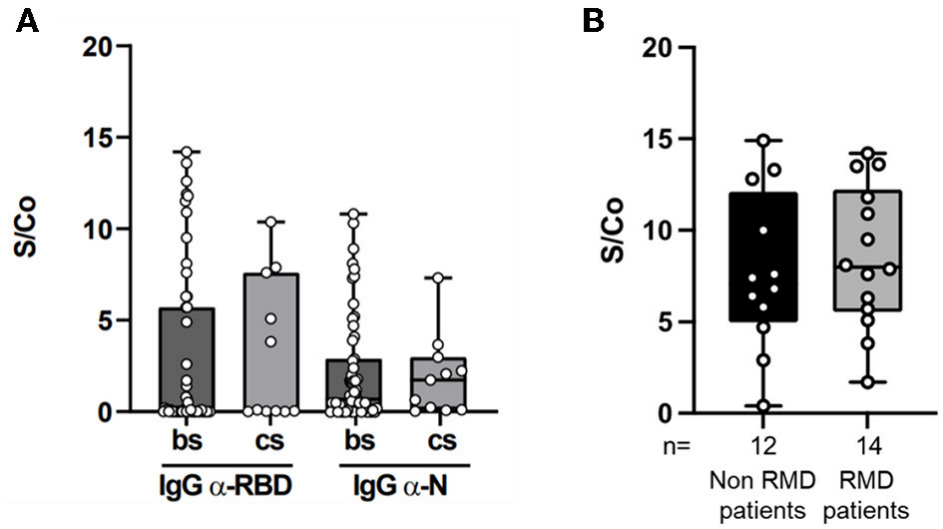
Magnitude of the anti-RBD and anti-N antibody response in RMD patients. **(A)** IgG levels against SARS-CoV-2 RBD (left panel) and N (right panel), expressed as Signal (S) vs. Control (Co) measured by ELISA in sera of RMD patients treated with b/ts-DMARD and cs-DMARD. **(B)** IgG levels against SARS-CoV-2 RBD in sera of RMD patients treated with b/ts-DMARD and cs-DMARD and in non-RMD patients not under 290 immunosuppressant treatment and RMD patients undergoing b/ts- or cs-DMARD treatment. Each dot into the box represent individual values, and bar min and max values. Statistical analyses were performed using Mann-Whitney *t*-test to compare two classes.

The magnitude of the antibody response to SARS-CoV-2 proteins in the 66 RMD seropositive patients, in terms of antibody levels against RBD and N proteins, was comparable between patients treated with b/ts-DMARDs and cs-DMARDs (Figure 1A), and it was not significantly impaired by concomitant treatment with GCs (data not shown, *p* = 0.56) (Figure 1A). Furthermore, the level of anti-RBD IgG titer in the RMD seropositive patients was similar to non-RMD control group (6.8 vs. 7.2 S/Co, respectively; *p* = 0.78) (Figure 1B). These data confirm the hypothesis that DMARDs treatment do not significantly impair the elicitation of antibody response.

### Factors Associated With SARS-CoV-2 Seropositivity

Factors associated with anti-RBD IgG positivity are shown in Table 3.

**Table 3:** IgG Anti-RBD SARS-CoV-2 seroprevalence and associated factors.

	**Total**	**IgG anti-RBD**		**IgG anti-RBD**		**OR**	**95% CI**		***P*-value**
		**positive cases**		**negative cases**					
	**(*n* = 358)**	**(*n* = 21)**	**(5.9%)**	**(*n* = 336)**	**(94.4%)**				
**Age group**									
<65 years	269	15	5.6%	254	94.4%	0.81	0.3032	2.1482	0.6809
≥ 65 years	89	6	6.7%	83	93.3%	1.22	0.4582	3.2445	1.2450
**Female**, ***n*** **(%)**	230	14	6.1%	216	93.9%	1.11	0.4365	2.8284	0.8363
**Diagnosis**									
RA	200	15	7.5%	185	92.5%	2.04	0.7729	5.3875	0.1503
SpA	158	6	3.8%	152	96.2%	0.48	0.1834	1.2784	0.1435
Rheumatoid factor	116	8	6.9%	108	93.1%	1.30	0.5229	3.2275	1.3126
anti-CPA	116	6	5.2%	110	94.8%	0.82	0.3104	2.1762	0.7060
**Anti-rheumatic treatment**									
b/tsDMARDs (%)	300	17	5.7%	283	94.3%	0.80	0.2576	2.4591	1.2447
bDMARDs (%)	278	16	5.8%	262	94.2%	0.90	0.3205	2.5490	0.8588
tsDMARDs (%)	22	1	4.5%	21	95.5%	0.75	0.0959	5.8634	1.1798
without csDMARDs	160	10	6.3%	150	93.8%	1.13	0.4662	2.7259	0.8024
csDMARDs association (%)	140	7	5.0%	133	95.0%	0.76	0.3001	1.9406	0.5821
csDMARDs monotherapy	58	4	6.9%	54	93.1%	1.23	0.3979	3.7941	1.2256
**PDN (%)–avarage dose**	105–4 mg	8–5.25 mg	7.6%	97–3.8 mg	92.4%	1.52	0.6092	3.7737	0.3709
≤ 2.5 mg	48	2	4.2%	46	95.8%	0.66	0.1496	2.9442	0.6018
>2.5 mg	57	6	10.5%	51	89.5%	2.24	0.8285	6.0311	0.1121
>10 mg	7	2	28.6%	5	71.4%	6.97	1.2682	38.2898	0.0253
At least 1 symptom	93	15	16.1%	78	83.9%	8.27	3.1034	22.0343	0.0000
Clinically suspected cases	38	7	18.4%	31	81.6%	4.92	1.8469	13.1028	0.0015
Contacts with COVID-19 cases	33	8	24.2%	25	75.8%	7.66	2.9008	20.2031	0.0000
**Comorbidities**									
At least 1	126	14	11.1%	112	88.9%	4.00	1.5700	10.1912	0.0037
>2	43	6	14.0%	37	86.0%	3.23	1.1814	8.8440	0.0222
CHD	14	0	0.0%	14	100.0%	0.00			
DM II	11	2	18.2%	9	81.8%	3.82	0.7718	18.9525	0.1002
Hypertension	89	11	12.4%	78	87.6%	3.64	1.4896	8.8872	0.0046
Obesity	32	6	18.8%	26	81.3%	4.77	1.7063	13.3305	0.0029
None	231	7	3.0%	224	97.0%	0.25	0.0981	0.6369	0.0037
Current smokers	79	1	1.3%	78	98.7%	0.17	0.0218	1.2521	0.0811

*RA, rheumatoid arthritis; SpA, spondyloarthritis; PDN, prednisone; csDMARDs, conventional synthetic disease-modifying anti-rheumatic drugs; b/tsDMARDs, biological/targeted synthetic disease-modifying anti-rheumatic drugs; TNF, tumor necrosis factor; IL, interleukin; CHD, coronary heart disease; DM II, diabetes mellitus type II*.

The highest rate of seropositivity was observed in patients classified as highly suspicious COVID-19 (18.4%; OR = 4.92, 95% CI = 1.84–13.1; *p* = 0.001) and those who had close contacts with confirmed COVID-19 cases (24.2%; OR = 7.66, 95% CI = 2.9–20.2; *p* < 0.0001). Seroprevalence was similar between woman and men (6.1 vs. 5.5%, *p* = ns) and was independent of age. No significant difference was identified between RA and SpA patients and anti-citrullinated protein antibodies (ACPA)/rheumatoid factor (RF) positivity did not influence the seroprevalence rate. An increased risk of seropositivity was observed in patients with at least one comorbidity (OR = 4.0, 95% CI = 1.57–10.19; *p* = 0.003), especially including hypertension (OR = 3.64, 95% CI = 1.48–8.88; *p* = 0.004) and obesity (OR = 4.77, 95% CI = 1.7–13.33; *p* = 0.003). The lack of comorbidities was associated with a significantly lower probability of anti-RBD positivity (OR = 0.25, 95% CI = 0.09–0.63; *p* = 0.004). Among ongoing anti-rheumatic drugs, no correlation emerged between anti-RBD positivity and therapy with conventional synthetic (including MTX), biological or targeted synthetic DMARDs. Conversely, GC therapy was associated with a dose-dependent progressive increase in the positivity rate resulting in a high OR for doses above 10 mg/day (OR = 6.97, 95% CI = 1.2–38.2, *p* = 0.02).

Finally, no factor has been found to correlate with the development of symptoms related to COVID-19 (Supplementary Table 2).

## Discussion

This seroprevalence study confirmed that the spread of SARS-CoV-2 infection among RMD patients was significantly wider than the prevalence estimated from the detection of symptomatic cases. In addition to the epidemiological results, the observation of a large proportion of asymptomatic cases and mild infections has great relevance with practical clinical implications, especially in the management of immunosuppressive therapy in this pandemic contest. In particular, no deaths or admissions to ICU were observed out of 66 subjects who tested positive for anti-SARS-CoV-2 antibodies in our cohort. These findings had not emerged from most of the studies conducted to date, which analyzed registry data of patients with confirmed COVID-19 only and selected mainly symptomatic infections with a more severe clinical course (15, 16). Furthermore, we have shown that both the seroprevalence of SARS-CoV-2 and the proportion of asymptomatic COVID-19 in RMD patients were consistent with that observed in the general population. Data from the seroprevalence survey on SARS-CoV-2 in Italy, conducted by ISTAT from 25th May to 15th July 2020, showed that the seroprevalence rate in the Italian general population (estimated on a sample of 64.660 persons) was 2.5% (95% CI 2.3–2.6) but reached a rate of 7.5% (95% CI 6.8–8.3%) in Lombardy, with maximum values recorded in the province of Bergamo (24%) and Cremona (19%). Our finding confirmed by a serological analysis what had already emerged in previous epidemiological studies conducted in the same and other geographical areas but limited by the lack of serological data on asymptomatic subjects (3–6). Other seroprevalence studies have been conducted worldwide during the first wave of the pandemic, but none of these was focused on the comparison between RMD and non-RMD patients (17–19). In addition, we demonstrated that the magnitude of the immune response in RMD patients was not significantly altered by different classes of DMARDs and it seemed to be comparable to a non-immunosuppressed control group.

Despite the theoretical fragility of RMD patients related to immunosuppressive therapies, our results showed that treatment with any kind of DMARD is not associated with an increased risk of SARS-CoV-2 infection. However, our serological analysis confirms the increased dose-dependent infectious risk associated with the chronic use of GCs (20), which is already known from several studies conducted on other types of infection and also emerged in a recent epidemiological analysis conducted by our group (21). Beyond the anti-rheumatic treatment, in our cohort the risk factors for SARS-CoV-2 infection were confirmed as those already known in the general population, namely the presence of comorbidities such as hypertension and obesity (22). The comparative analysis and the search for factors associated with SARS-CoV-2 seropositivity was conducted only on anti-RBD IgG which has been shown to provide the best specificity for SARS-CoV-2 and appear to decrease more slowly over time than levels of other classes of antibody (23, 24). IgM and IgA antibodies have been included to define the seropositivity rate but no comparative analysis has been performed on these antibody classes. However, we found that patients who tested positive to IgM or IgA and negative to IgG had higher rate of asymptomatic infections than IgG positive patients. Since both IgM and IgA have been shown to appear in the first 3 to 4 days of SARS-CoV-2 infection (25), our hypothesis is that we identified patients in the very early phase of COVID-19, before they developed symptoms or even seroconverted to IgG.

In conclusion, given the still pressing need to optimize the management of RMD patients during the pandemic and to organize their prioritization of access to the vaccine programme, the results of this study are reassuring about the low impact of RMDs and immunomodulatory therapies on the risk and clinical course of COVID-19. Our results provide a further contribution to the management strategy of RMD patients, in the direction of reaffirming the importance of maintaining ongoing anti-rheumatic therapy even during the most critical phases of the pandemic.

Moreover, the antibody titer measured in RMD patients, which was comparable to that of the control population, suggests an adequate humoral response to SARS-CoV-2 infection and, presumably, to vaccination.

## Data Availability Statement

The original contributions presented in the study are included in the article/Supplementary Material, further inquiries can be directed to the corresponding author.

## Ethics Statement

The studies involving human participants were reviewed and approved by Ethics Committee Milano Area 2. The patients/participants provided their written informed consent to participate in this study.

## Author Contributions

EF, MBi, and GM collected clinical data and blood samples. EF and GM drafted the manuscript. MBo, EP, AF, TF, and MM contributed to experimental data and related analysis. AG made data elaboration and statistical analysis. EM provided the ELISA kits for analysis. RG, SA, and RC drafted and revised the manuscript. All authors contributed to the article and approved the submitted version.

## Funding

The project was co-financed by Lombardy 2014–2020 Operational Program under the European Regional Development Fund (MAINSTREAM project).

## Conflict of Interest

EM is employed by Dia.Pro, Diagnostic Bioprobes Srl. The remaining authors declare that the research was conducted in the absence of any commercial or financial relationships that could be construed as a potential conflict of interest.

## Publisher's Note

All claims expressed in this article are solely those of the authors and do not necessarily represent those of their affiliated organizations, or those of the publisher, the editors and the reviewers. Any product that may be evaluated in this article, or claim that may be made by its manufacturer, is not guaranteed or endorsed by the publisher.
