# Corrigendum: The Impact of Anti-rheumatic Drugs on the Seroprevalence of Anti-SARS-CoV-2 Antibodies in a Cohort of Patients With Inflammatory Arthritis: The MAINSTREAM Study

**DOI:** 10.3389/fmed.2022.923790

**Published:** 2022-05-04

**Authors:** Ennio Giulio Favalli, Andrea Gobbini, Mauro Bombaci, Gabriella Maioli, Martina Biggioggero, Elisa Pesce, Andrea Favalli, Martina Martinovic, Tanya Fabbris, Edoardo Marchisio, Alessandra Bandera, Andrea Gori, Sergio Abrignani, Renata Grifantini, Roberto Caporali

**Affiliations:** ^1^Division of Clinical Rheumatology, ASST Gaetano Pini-CTO Institute, Milan, Italy; ^2^Department of Clinical Sciences and Community Health, Research Center for Adult and Pediatric Rheumatic Diseases, Università degli Studi di Milano, Milan, Italy; ^3^Istituto Nazionale Genetica Molecolare, Padiglione Romeo ed Enrica Invernizzi, Milan, Italy; ^4^Dia.Pro, Diagnostic Bioprobes Srl, Milan, Italy; ^5^Infectious Diseases Unit, Fondazione IRCCS Ca' Granda Ospedale Maggiore Policlinico, Milan, Italy; ^6^Department of Pathophysiology and Transplantation, Università degli Studi di Milano, Milan, Italy; ^7^Centre for Multidisciplinary Research in Health Science (MACH), Università degli Studi di Milano, Milan, Italy

**Keywords:** seroprevalence, SARS-CoV-2, humoral response, rheumatic musculoskeletal diseases, disease-modifying anti-rheumatic drugs, risk of infection, COVID-19

In the original article, an author name was incorrectly spelled as “Alessandra Bandiera.” The correct spelling is “Alessandra Bandera.”

The authors apologize for this error and state that this does not change the scientific conclusions of the article in any way. The original article has been updated.

## Publisher's Note

All claims expressed in this article are solely those of the authors and do not necessarily represent those of their affiliated organizations, or those of the publisher, the editors and the reviewers. Any product that may be evaluated in this article, or claim that may be made by its manufacturer, is not guaranteed or endorsed by the publisher.

