# Supplementary material for: The Impact of Anti-rheumatic Drugs on the Seroprevalence of Anti-SARS-CoV-2 Antibodies in a Cohort of Patients With Inflammatory Arthritis: The MAINSTREAM Study
Source: Front Med (Lausanne). 2022 Mar 11;9:850858. doi: 10.3389/fmed.2022.850858 (PMC8963104; doi:10.3389/fmed.2022.850858)
Supplement: Supplementary file 1 [file Data_Sheet_1.docx]

**Supplementary Table 1.** Classification criteria of symptoms

**Supplementary Figure 1.** Clinical manifestations of RMD patients.

**Supplementary table 2**. Factors associated with COVID-19 symptoms development

|  | **Positive cases**  **n = 66** | **Positive symptomatic cases**  **n = 27** | **Positivi asimptomatic cases**  **n = 38** | **OR** | **95 % CI** | **P value** |
| --- | --- | --- | --- | --- | --- | --- |
| Age, mean (SD), years | 55 (13.9) | 50 (13.4) | 55 (13.5) |  |  |  |
| **Age group**  19-43 (%)  44-53 (%)  54-63 (%)  64-83 (%) | 17  12  16  20 | 7 (41.2)  7 (58.3)  8 (50)  5 (25) | 10 (58.9)  5 (41.6)  8 (50)  15 (75) | 0.98  2.31  1.57  0.34 | 0.3186-3.0141  0.6455-8.2668  0.507-4.9176  0.1083-1.1215 | 0.97  0.1981  0.4307  0.0771 |
| **Female, n (%)** | 41 | 19 (46.3) | 22 (55.7) | 1.7273 | 0.606-4.9233 | 0.3064 |
| **Diagnosis** |  |  |  |  |  |  |
| RA  SpA | 36  29 | 15 (41.7)  12 (41.4) | 21 (58.3)  14 (58.6) | 1.0119  1.3714 | 0.375-2.7302  0.5018-3.7479 | 0.9814  0.5380 |
| **Anti-rheumatic treatment** |  |  |  |  |  |  |
| **b/tsDMARDs (%)**  **mono**  **csDMARDs association (%)** | 55  35  20 | 24 (43.6) 14 (40) 10 (50) | 31 (56.3) 21 (60) 10 (50) | 1.8065  0.8718 1.6471 | 0.4222-7.7299 0.3241-2.3447 0.5685-4.7717 | 0.4253  0.7858  0.3579 |
| **bDMARDs (%)** | 51 | 22 (43.2) | 29 (56.8) | 1.3655 | 0.4009-4.6515 | 0.6184 |
| **tsDMARDs (%)** | 4 | 2 (50) | 2 (50) | 1.44 | 0.1900-10.9132 | 0.7242 |
| **csDMARDs mono (%)** | 10 | 3 (30) | 7 (70) | 0.5536 | 0.1294-2.3688 | 0.4253 |
| **PDN (%) – avarage dose**  **≤2.5 mg**  **>2.5 mg** | 19 – 4.2 mg  7  12 | 8 (42)  1 (14.3)  7 (58.3) | 11 (58)  6 (85.7)  5 (41.7) | 1.0335  0.2051  2.31 | 0.3498-3.0539  0.0232-1.8135  0.6455-8.2668 | 0.9525  0.1543  0.1918 |
| **MTX**  **noMTX**  **Combo**  **mono** | 22  43  13  9 | 8 (36.4)  19 (44.2)  6 (46.2)  2 (22.2) | 14 (63.6)  24 (55.8)  7 (53.8)  7 (77.8) | 0.7218  1.3854  1.2653  0.3543 | 0.2509-2.0768  0.4815-3.9861  0.3724-4.2990  0.0675-1.8586 | 0.5454  0.5454  0.7061  0.2198 |
| RA rheumatoid arthritis, SpA spondyloarthritis, PDN prednisone, csDMARDs conventional synthetic disease-modifying anti-rheumatic drugs, b/tsDMARDs biological/targeted synthetic disease-modifying anti-rheumatic drugs, TNF tumor necrosis factor, IL interleukinMTX methotrexate. | | | | | | |
